# The Roles of Various Prostaglandins in Fibrosis: A Review

**DOI:** 10.3390/biom11060789

**Published:** 2021-05-24

**Authors:** Ke Li, Jing Zhao, Mingxuan Wang, Lingzhi Niu, Yuanping Wang, Yanxia Li, Yajuan Zheng

**Affiliations:** Department of Ophthalmology, The Second Hospital of Jilin University, Changchun 130000, China; like19@mails.jlu.edu.cn (K.L.); lhbswqw@126.com (J.Z.); wangmx17@mails.jlu.edu.cn (M.W.); niulz18@mails.jlu.edu.cn (L.N.); wyp19@mails.jlu.edu.cn (Y.W.); lyx20@mails.jlu.edu.cn (Y.L.)

**Keywords:** fibrosis, myofibroblast, PGE2, PGD2, PGI2, PGF2α, TXA2

## Abstract

Organ fibrosis is a common pathological result of various chronic diseases with multiple causes. Fibrosis is characterized by the excessive deposition of extracellular matrix and eventually leads to the destruction of the tissue structure and impaired organ function. Prostaglandins are produced by arachidonic acid through cyclooxygenases and various prostaglandin-specific synthases. Prostaglandins bind to homologous receptors on adjacent tissue cells in an autocrine or paracrine manner and participate in the regulation of a series of physiological or pathological processes, including fibrosis. This review summarizes the properties, synthesis, and degradation of various prostaglandins, as well as the roles of these prostaglandins and their receptors in fibrosis in multiple models to reveal the clinical significance of prostaglandins and their receptors in the treatment of fibrosis.

## 1. Introduction

Organ fibrosis is a common pathological result of chronic tissue damage caused by various etiological factors. This condition is often defined as a degenerative process of connective tissue that is accompanied by the excessive formation and deposition of extracellular matrix (ECM) components, resulting in the destruction of normal organ architecture and function [1]. Fibrotic responses share the same initial fundamental mechanism as the normal wound healing process: the generation of new tissue to replace damaged tissue. However, when this process exceeds that of normal repair, it will cause pathological fibrosis with the accumulation of nonfunctional scarring and destruction of the normal tissue architecture [2]. The pathogenesis of fibrosis is associated with various diseases, including idiopathic pulmonary fibrosis (IPF), heart failure, liver cirrhosis, nonalcoholic steatohepatitis, chronic kidney disease, scleroderma, and glaucoma. However, no effective treatments are available to prevent or reverse this process. Nintedanib and pirfenidone are the only two drugs approved by the Food and Drug Administration to treat IPF [3] but only retard disease progression. Therefore, a better understanding of the processes and mechanisms of fibrosis will help identify more specific and efficient strategies to reduce the morbidity and mortality caused by fibrosis. Prostaglandins (PGs) are lipid mediators that participate in various physiological reactions. Multiple studies have indicated that PGs also play an important role in fibrosis. The purpose of this review is to summarize the key biological features of various PGs and to discuss their roles in fibrotic processes.

## 2. Comprehensive Mechanisms of Fibrosis

Fibrosis is a complex process that requires multiple cells and active mediators. Continuous tissue damage or exposure to harmful substances induces a local inflammatory response by activating immunocytes (macrophages, dendritic cells, and mast cells), the complement system and the coagulation/fibrinolysis systems and inducing the secretion of various biological inflammatory mediators. These changes all induce typical inflammatory signs, including redness, swelling, heat, pain, and impairment or loss of function, on the one hand, and clear production of harmful or damaged material on the other hand [2]. This inflammatory process is an effective mechanism by which the body reduces damage and protects organ function. However, if the removal is not complete, a persistent inflammatory response with immune cell accumulation and further cytokine and enzyme release will lead to the death of parenchymal cells and uncontrolled production and activation of profibrotic cytokines, such as transforming growth factor-β (TGF-β), connective tissue growth factor (CTGF), and platelet-derived growth factor (PDGF), which in turn activate various progenitor cells, transforming them into myofibroblasts with high expression of alpha-smooth muscle actin (α-SMA), increasing cell proliferation and production of ECM and decreasing myofibroblast apoptosis. These cells finally drive pathological fibrosis [2,4].

Myofibroblasts are an important cellular component that produce ECM and promote tissue fibrosis and were first detected in the granulation tissue of healing skin wounds as the final differentiated form of fibroblasts [5]. Different mechanisms, including cellular activation, transformation, proliferation, infiltration, expansion, epithelial-to-mesenchymal transition (EMT), mesothelial-to-mesenchymal transition (MMT), and endothelial-to-mesenchymal transition (EndoMT), are involved in increasing the number of myofibroblasts [2]. Myofibroblasts, which express α-SMA, not only have secretory functions similar to those of fibroblasts but also possess ultrastructural and physiological characteristics similar to those of smooth muscle cells; hence, they rapidly induce the production and stimulation of ECM and contract the ECM via stress fibers, resulting in the deformation of the tissue structure and scar formation [6,7]. As discussed previously, TGF-β not only induces ECM formation in profibrogenic cells but also acts as the key factor inducing the activation of myofibroblasts. Therefore, inhibiting myofibroblast activation through the above mechanisms is also an effective strategy to prevent or reverse fibrosis.

## 3. Production of Prostaglandins

PGs, which are secretory lipid mediators generated from arachidonic acid (AA), play an important role in regulating various biological functions in humans. These molecules are members of a subclass of eicosanoids containing C20 atoms with a cyclopentane (5-carbon) ring and are divided into prostacyclopentanes and thromboxanes, depending on their structures [8].

AA is a type of polyunsaturated fatty acid that exists as a membrane phospholipid on cells. AA is released through phospholipid hydrolysis by the phospholipase A2 (PLA2), phospholipase D, or phospholipase C pathways in response to cytokines, growth factors, and other proinflammatory stimuli [9] and then is converted to PGs and leukotrienes by the cyclooxygenase (COX) and lipoxygenase (LOX) pathways, respectively. COX, also called PTGS or PGHS, catalyzes cyclooxygenase and endoperoxidase reactions, leading to the production of PGH2 from AA via PGG2. The generated PGH2 can be converted to PGE2, PGD2, PGI2, PGF2α, and thromboxane A2 (TXA2) by individual enzymes and isomerases [10], including PGE synthase (PGES), PGD synthase (PGDS), PGI synthase (PGIS), PGF synthase (PGFS), and TXA synthase (TXAS), respectively [8].

A series of G protein-coupled rhodopsin-type receptors located on the surface of target cells mediate the function of these PGs and consist of eight types: prostaglandin D receptor (DP1), prostaglandin E receptors (EP1, EP2, EP3, and EP4), prostaglandin F receptor (FP), prostaglandin I receptor (IP), and thromboxane receptor (TP). In addition, another G protein-coupled receptor termed chemoattractant receptor-homologous molecule is expressed on T helper 2 cells (CRTH2 or DP2) and responds to PGD2 but belongs to the superfamily of N-formyl-methionyl-leucyl-phenylalanine chemoattractant receptors.

**Figure 1:**
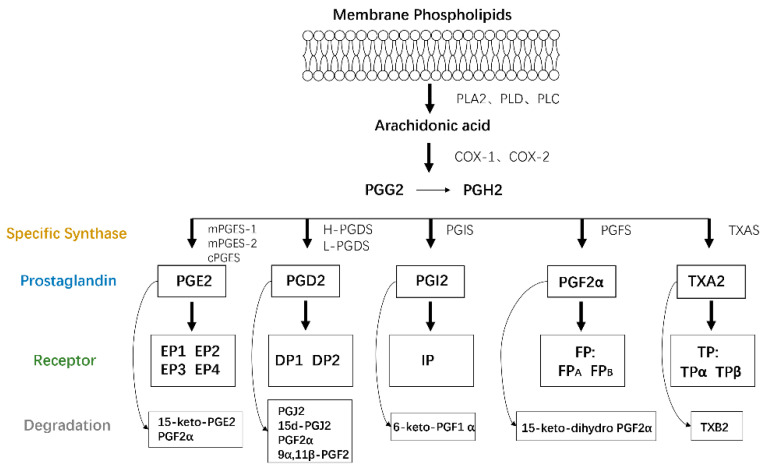
PG production. Membrane phospholipids are metabolized into AA by the PLA2, PLD, or PLC pathways. AA is then converted to PGs and IL by the COX and LOX pathways, respectively. The generated PGH2 is transformed to PGE2, PGD2, PGI2, PGF2α, and thromboxane A2 (TXA2) by individual enzymes, all of which function by binding to target receptors on their own cells or adjacent cells in an autocrine or paracrine manner.

A dynamic balance between PG production and degradation is needed to maintain physiological homeostasis. PGs are primarily metabolized by the initial oxidation of the 15(S)-hydroxyl group catalyzed by 15-hydroxyprostaglandin dehydrogenases (15-PGDHs), which are comprised of two types, type I NAD+-dependent 15-PGDH and type II NADP-dependent 15-PGDH, and type I is considered the key enzyme involved in controlling the biological activities of PGs and related eicosanoids [11]. Due to the role of PGs in fibrosis, the pathological process of fibrosis is also indirectly altered by the regulation of 15-PGDH expression. The specific details will be discussed later (Figure 1).

## 4. Prostaglandins

PGs are expressed in almost all cell types and perform various functions, such as maintaining the physiological balance, regulating inflammation, and participating in tumor formation or migration, in an autocrine or paracrine manner. However, some studies have found that PGs also play multiple roles in tissue fibrosis, which involves several cell types, such as fibroblasts, alveolar epithelial cells, renal mesangial cells, and hepatic stellate cells (HSCs). Next, we summarize the roles of all PGs in fibrosis and the possible mechanisms, which will provide new information to help elucidate fibrotic pathogenesis or therapeutic targets (Table 1).

### 4.1. PGE2

PGE2 is one of the most abundant PGs produced in the body and exhibits versatile biological activities. On the one hand, PGE2 is vital for many biological functions under physiological conditions, such as the regulation of immune responses, smooth muscle contraction/dilation, gastrointestinal integrity, sodium homeostasis, and fertility. On the other hand, dysregulated or uncontrolled PGE2 synthesis or degradation is associated with a wide range of pathological conditions, including chronic inflammation, Alzheimer's disease, and tumorigenesis [12,13]. In addition, PGE2 participates in the pathological fibrotic process in various cells or tissues through a series of signaling pathways.

#### 4.1.1. Production of PGE2

PGE2 is synthesized from AA by COX and specific PGESs, including microsomal PGE synthase-1 (mPGES-1), microsomal PGE synthase-2 (mPGES-2), and cytosolic PGE synthase (cPGES). mPGES-1 is a membrane-associated protein in the eicosanoid and glutathione metabolism (MAPEG) family that is constitutively expressed at low levels under homeostatic conditions; however, mPGES-1 is substantially upregulated in response to various inflammatory stimuli and is responsible for the production of PGE2 in inflammation specifically coupled to COX-2 [14]. Unlike mPGES-1, mPGES-2 is constitutively expressed in many cells and tissues and is not induced by inflammatory stimuli. In vitro studies have found that mPGES-2 exerts its PGE2 synthase activity via both COX-1 and COX-2 in immediate and delayed responses, with a modest COX-2 preference [15]. However, in vivo studies have revealed that the production of PGE2 in mPGES-2 gene-deficient mouse tissues and cells does not change [16]. Consistent with this finding, Fusao found that mPGES-2 only catalyzes PGE2 synthesis in the heme-free form in vitro, while in vivo, it does not change PGE2 production as a heme-bound protein [17]. cPGES is localized in the cytoplasmic compartment, is constitutively expressed in a wide variety of mammalian cell lines and tissues, and converts PGH2 to PGE2 in association with COX-1, particularly during the immediate PGE2 biosynthetic response elicited by Ca^2+^-evoked stimuli [13,18]. Therefore, mPGES-1 is the primary enzyme catalyzing PGE2 production, which has been shown to regulate the fibrotic response. mPGES-1 exerts an essential effect on pulmonary fibrogenesis in mice via EP2-mediated signal transduction, and activation of mPGES-1/PGE2/EP2/focal adhesion kinase signaling may represent a new therapeutic strategy for the treatment of patients with IPF [19]. An mPGES-1 deficiency in a mouse model of nonalcoholic steatohepatitis with decreased PGE2 production augmented the TNF-α-triggered inflammatory response and hepatocyte apoptosis [20]. mPGES-1 also protects against renal fibrosis and inflammation during obstructive nephropathy via the mPGES-1/PGE2/EP4 pathway [21].

After PGE2 is formed, it is transported through the membrane by ATP-dependent multidrug resistance protein-4 or diffuses across the plasma membrane to act at or near its site of secretion. PGE2 signals through four receptors, EP1, EP2, EP3, and EP4. EP1 coupled with Gq mediates phospholipase C activation, resulting in the accumulation of inositol 1,4,5-trisphosphate (IP3) and diacylglycerol (DAG), which in turn induce Ca^2+^ release from the endoplasmic reticulum and activate protein kinase C (PKC). EP2 and EP4 couple to Gs to increase the intracellular concentration of cyclic adenosine monophosphate (cAMP), which subsequently activates protein kinase A (PKA). However, EP3 plays a unique role, with multiple splice variants defined by unique C-terminal cytoplasmic tails. EP3 isoforms couple to Gi or G12 to increase the concentration of intracellular Ca^2+^, inhibit cAMP generation and activate the small G protein Rho [10,13,18,22]. Therefore, the functions of PGE2 mainly depend on the type and proportion of activated receptors in the tissue or cells. An article analyzing the expression of various PG receptors in human lung fibroblasts from normal individuals and patients with IPF via RNA sequencing and western blotting revealed the prominent expression of EP2, with lower expression of EP4 receptors and barely discernible expression of EP1 or EP3 in normal fibroblasts. Moreover, the expression of EP2 and EP4 decreased in fibroblasts from patients with IPF [23], indicating that EP2 and EP4 may be involved in the response to pulmonary fibrosis.

#### 4.1.2. Effect of PGE2 on Fibrosis

The role of PGE2 in fibrosis is complex and may be related to the receptor types, target cell types, and organs. The protective effect on fibrosis by acting on multiple cells via EP2 and EP4 receptors. PGE2 is produced by various cells in different tissues, including alveolar epithelial cells, tubular epithelial cells, fibroblasts, and alveolar macrophages. The secreted PGE2 acts on fibroblasts or epithelial cells in an autocrine or paracrine manner, thus disrupting the process of tissue fibrosis [24]. Many studies have shown that PGE2 exerts antifibrotic effects on different organs by inhibiting cell proliferation [25,26], migration [27], collagen expression and deposition [25,28,29], and fibroblast differentiation [30,31] by activating the cAMP/PKA signaling pathway upon binding to EP2 or EP4 in fibroblasts. Inhalation of liposome-coated PGE2 in the lungs significantly improves the symptoms of bleomycin-induced pulmonary fibrosis in mice, including weight loss and the reduction in fibrosis-related gene expression, and improves the survival rate of animals [32].

##### Changes in PGE2 Expression in Different Organs

PGE2 expression also changes in different fibrotic diseases. Notably, PGE2 is normally present at high concentrations in respiratory epithelial lining fluid (ELF), where it suppresses mesenchymal cell proliferation mediated by polypeptide-derived growth factors. However, fibroblasts derived from the lungs of patients with IPF and systemic sclerosis (SSc) produce low levels of PGE2, and the levels in the ELF of patients with IPF were also found to be 50% lower than normal [33,34]. This finding may result from the combination of decreased COX-2 expression in lung fibroblasts and increased 15-PGDH expression in the intact alveolar structures preserved in lung tissues of patients with IPF [30,35]. The limited capacity of fibrotic lung fibroblasts to upregulate COX-2 expression may be due to epigenetic regulation of the COX-2 promoter region, such as hypermethylation of the transcriptional regulator chromosome 8 open reading frame 4 (c8orf4) [34], H3 and H4 histone deacetylation, and H3K9 and H3K27 methylation [36]. In the mouse model of hepatic fibrosis induced by CCl_4_, PGE2 levels in the liver also decreased significantly [37]. Unlike lung tissue, the EP2 mRNA is expressed at low levels in the kidneys under physiological conditions [38], but the expression of the COX-2, EP2, and EP4 mRNAs increased significantly after unilateral ureteral obstruction (UUO), which were detected mainly in tubular epithelial cells and interstitial cells [39,40]. 

##### Regulation of PGE2 in Cell Proliferation and Apoptosis

Interstitial cells and parenchymal cells play an important role in the development of pulmonary fibrosis, and PGE2 affects the survival and apoptosis of these cells to prevent fibrotic diseases. First, in the lung, exogenous PGE2 inhibits the proliferation of patient-derived normal lung fibroblasts via EP2 receptor and cAMP activation [25]. PKA is the classic effector of cAMP and is responsible for cell growth and differentiation. However, the inhibitory effect of PGE2 on proliferation is mediated by another effector named exchange protein activated by cAMP-1 (Epac-1) through the activation of the small GTPase Rap1, and PKA activation is mainly responsible for regulating collagen expression [41]. In addition, PGE2 also inhibits the FGF-induced expression of a number of cell cycle genes, including CCND1, CCNB1, and PLK1, which results from the brake for the binding between the transcription factor Forkhead box M1 (FOXM1) and above target gene-promoter elements of human lung fibroblast [42]. Under normal circumstances, timely apoptosis of fibroblasts avoids the pathological changes of fibrosis, caused by its excessive accumulation. However, some studies have found that fibroblasts from patients with pulmonary fibrosis are resistant to apoptosis. However, PGE2 restores the sensitivity of fibroblasts to apoptosis and promotes apoptosis by activating the EP2/EP4 receptor through an increase in phosphatase and tensin homolog on chromosome ten (PTEN) activity, decrease in Akt activity and increase in Fas receptor expression [43]. Consistent with the findings described above, PGE2 also regulates the survival and apoptosis of renal cells. PGE2 produced by renal tubular cells inhibits proliferation and induces the apoptosis of interstitial fibroblasts in a paracrine manner and improves the survival and regeneration of tubular cells in an autocrine manner via EP4, and this result may be related to the reduced production of chemokines related to inflammatory infiltrates [39,44]. Several studies have also revealed that the expression of COX-2/PGE2 decreases the apoptosis of hepatocytes but increases the apoptosis and inactivation of HSCs with inhibiting the proliferation by downregulating miR-23a-5p and miR-28a-5p expression in HSCs [45]. In addition to affecting interstitial cell and parenchymal cell survival, PGE2 alters the migration of fibroblasts by increasing PTEN levels in the heart, which is another important feature of wound repair at the site of injury [46].

##### Regulation of PGE2 in Myofibroblast Differentiation

Myofibroblast differentiation induced by mediators, such as TGF-β and biomechanical signals, is an important step in the pathological progression of fibrosis. These myofibroblasts not only have stronger proliferative and migratory abilities but also exhibit increased synthesis and secretion of ECM. PGE2 not only inhibits but also reverses myofibroblast differentiation. Through microarrays, PGE2 was shown to reverse the changes in gene expression induced by TGF-β1. Genes upregulated by TGF-β1 and downregulated by PGE2 tend to be associated with cell adhesion, contractile fibers, and actin binding, whereas genes downregulated by TGF-β1 but subsequently upregulated by PGE2 are enriched in glycoprotein, polysaccharide binding, and regulation of cell migration [47]. α-SMA is a marker of myofibroblast differentiation, and the transcription factors serum response factor (SRF) and myocardin-related transcription factor-A (MRTF-A) are important for regulating α-SMA expression. PGE2 inhibits the expression of SRF by inhibiting P38 activation and inhibits the nuclear import of MRTF-A via the activation of cofilin 1 and inactivation of vasodilator-stimulated phosphoprotein, thus reducing the formation of nuclear MRTF-A-SRF complexes and subsequently inhibiting α-SMA promoter activation in normal lung fibroblasts [48]. Myofibroblasts were previously considered terminally differentiated cells, but with the development of research sites, myofibroblasts were also shown to have the ability to dedifferentiate and are characterized by the disappearance of α-SMA. Therefore, approaches promoting the dedifferentiation of myofibroblasts are also necessary for fibrosis resolution. As discussed in a previous study, undifferentiated fibroblasts appear spindle-shaped and elongated, in stark contrast to the larger, cuboidal, and stellate-shaped myofibroblasts. Myofibroblasts exposed to PGE2 appear smaller, thinner, and display fewer cytoplasmic projections, along with the downregulation of α-SMA, eradication of stress fibers and reduction in ECM production, which is mediated by the EP2/cAMP/PKA pathway [49]. As research has progressed, more mechanisms involved in fibrosis have been discovered. Ca^2+^ oscillations induced by TGF-β are sufficient to increase the production of ECM proteins. The inhibitory effect of PGE2 on the expression of ECM genes and conversion of fibroblasts to a myofibroblast phenotype appears to occur via cAMP generated by signaling from the EP2 receptor and apparently the EP4 receptor, which blunts Ca^2+^ oscillations promoted by TGF-**β** or present in HPFs from patients with IPF and inhibits the activation of Ca^2+^/calmodulin-dependent protein kinase-II (CaMK-II) [23]. In renal tissue, EP2 receptor stimulation reduces TGF-β1-induced injury and fibrosis in mouse mesangial cells (MCs) by decreasing endoplasmic reticulum stress and transient receptor potential cation channel protein (TRPC) via the inhibition of excessive ERK signaling [50].

PGE2 exerts an antifibrotic effect by activating EP2 or EP4 receptors, and various agonists of this receptor have also been used in antifibrotic studies. The EP2 receptor agonist butaprost inhibits renal fibrosis and reduces the expression of α-SMA, fibronectin, and col1 in Madin-Darby canine kidney (MDCK) cells, a mouse model of unilateral ureteral obstruction and human precision-cut kidney slices, but this effect is not achieved through the activation of the cAMP/PKA signaling pathway but through the inhibition of the TGF-β/Smad signaling pathway [38,40]. The EP4 agonist ONO-0260164 also exerts antifibrotic effects on cardiac fibrosis via the downregulation of collagen type 1 and type 3 in vivo and in vitro through PKA activation [51]. However, the damage caused by EP4 agonists to glomerular tissue limits their clinical application. In cultured renal fibroblasts isolated from WT kidneys, ONO-AE1-329 significantly suppresses PDGF-BB-induced proliferation in a concentration-dependent manner [39]

However, the antifibrotic effect of PGE2 is controversial. Although the presence of PGE2 inhibits the proliferation, transformation, and ECM production of fibroblasts in most cases, these effects are mainly mediated by EP2/EP4 receptors. The EP1/EP3 receptor-coupled signal is associated with promoting fibrosis due to the differences in G proteins coupled with the EP1/EP3 receptors and the differences in downstream cAMP and Ca^2+^ regulation. Activation of EP1/EP3 receptors by PGE2 may serve to induce the proliferation of MCs and cardiac fibroblasts and promote the accumulation of ECM, effects that are mediated by the stimulation of cyclin D1 with involvement of both the p42/44 MAP kinase pathway and the PI3 kinase pathway [52,53] or the induction of excessive ERK signaling [50].

Overall, PGE2 participates in the fibrosis of various organs and tissues by regulating all hallmarks of profibrotic fibroblasts induced by TGF-β through the activation of the corresponding receptors. However, the specific effect of PGE2 is context-specific and cell/receptor type-dependent. Therefore, the fibrotic effects of PGE2 on various organs must be studied separately and generally cannot be defined. The promotion of endogenous PGE2 generation or inhibition of endogenous PGE2 degradation by external stimuli is a potentially useful method for the treatment of fibrosis to avoid defects in PGE2 chemical instability and for greater efficacy, which has strong prospects for clinical application.

### 4.2. PGD2

PGD2 is a major lipid mediator with physiological effects on both the peripheral nervous system and central nervous system (CNS) [13]; it regulates vasodilatation, bronchoconstriction, platelet aggregation, glycogenolysis, allergic reactions, and a reduction in intraocular pressure in peripheral tissues and modulates sleep induction, body temperature, olfactory function, nociception, and neuromodulation in the CNS [54].

#### 4.2.1. Production of PGD2

Two enzymes are responsible for the synthesis of PGD2 from PGH2: hematopoietic-type PGD synthases (H-PGDS) and lipocalin-type PGD synthases (L-PGDS). H-PGDS mediates the production of PGD2 in mast cells and other hematopoietic cells, while L-PGDS is expressed in oligodendrocytes, the choroid plexus, organs of the male genital tract, leptomeninges, and hearts of humans and monkeys [10].

PGD2 usually regulates physiological functions through its specific receptors DP1 and DP2 [also known as chemoattractant receptor homologous molecule expressed on TH2 lymphocytes (CRTH2)] [55]. Multiple tissues and cells, such as nasal serous glands, the vascular endothelium, Th2 cells, dendritic cells, basophils, and eosinophils, express the DP1 receptor [10]. Similar to EP2 or EP4, DP1 receptor activation leads to increased cAMP levels and intracellular PKA activation. CRTH2 is mainly expressed in Th2 cells and couples to the Gi protein to inhibit cAMP synthesis and increase intracellular Ca^2+^ concentrations. This finding suggests that PGD2 interferes with the fibrotic process by activating its receptor.

PGD2 is a relatively unstable lipid with a half-life of approximately 30 min in plasma and can be metabolized to other types, including PGF2α, 9α,11β-PGF2 and the J series of PGs (such as PGJ2, Δ12-PGJ2, and 15d-PGJ2) [13]. Moreover, 15d-PGJ2, a natural ligand that activates peroxisome proliferator-activated receptor (PPAR-γ), inhibits the NF-κB pathway and induces oxidative stress, is an important lipid participating in various biological and pathological conditions [56,57]. A stereoisomer of PGF2α, 9α,11β-PGF2, which is metabolized from PGD2 by the enzyme PGD 11-ketoreductase, has been shown to mediate various biological activities, such as the contraction of bronchial smooth muscle cells, inhibition of platelet aggregation and induction of chemoattraction of various immune cells [56].

#### 4.2.2. Effect of PGD2 on Fibrosis

The function of PGD2 in the inflammatory response is complex and not only promotes the development of inflammation by stimulating the chemotaxis of eosinophils, basophils, and Th2 lymphocytes but also inhibits the activation of inflammatory cells such as antigen-specific T cells and basophils [58]. H-PGDS knockout mice exhibit aggravated bleomycin-induced collagen deposition in the lung, accompanied by the early accumulation of inflammatory cells and inflammatory cytokines and increased vascular permeability [58]. H-PGDS is expressed at high levels by monocyte macrophages and neutrophils in a bleomycin-induced mouse model of pulmonary fibrosis, while it is also expressed in epithelial cells and vascular endothelial cells in an endotoxin-induced inflammation model. PGD2 derived from these cells reduces the inflammatory and fibrosis responses of lung tissue by inhibiting the aggregation of inflammatory cells and reducing vascular permeability [58]. Therefore, PGD2 plays an important role in the pathological fibrotic process in some organs.

In the lung, PGD2 induces antifibrotic effects by inhibiting TGF-β-induced collagen secretion and fibroblast proliferation via the activation of the DP receptor and suppression of early inflammation [58,59,60], which is achieved by cAMP accumulation [59]. However, CRTH2, another PGD2 receptor expressed in Th2 group 2 cells, innate lymphoid cells, eosinophils, and basophils, is also vital for the inhibition of fibrosis. The absence of CRTH2 exacerbates bleomycin-induced pulmonary inflammation and fibrosis in mice, changes that are alleviated by the transfer of wild-type splenocytes, especially γδT cells expressing CRTH2, by inducing the expression of the anti-fibrosis factor IL-10 [61]. In the liver, PGD2 inhibits VEGF expression induced by TGF-β in HSCs from chronic schistosome granulomas, indicating that PGD2 may be a novel target for the treatment of schistosomal hepatic granuloma [62].

Both PGD2 and its metabolite inhibit fibrosis. An increasing body of in vivo or in vitro evidence has shown that 15d-PGJ2 possesses antifibrotic properties in various experimental models, most of which occur in a PPAR-γ-independent manner. Alon et al. showed that 15d-PGJ2 in keloids attenuates keloid cell proliferation, inhibits collagen gel contraction, and increases cell apoptosis by inducing oxidative stress in vitro [56]. AKR1C3 is an enzyme that metabolizes PGD2 to 9α,11 β-PGF2, the inhibition of which increases the concentration of 15d-PGJ2 and is a potential treatment for skin keloids [56]. P38 mitogen-activated protein kinase (MAPK) is an important downstream molecule of the TGF-β signaling pathway that mediates fibrosis. Kye-Im et al. found that 15d-PGJ2 inhibits cat corneal myofibroblast transformation and ECM production by decreasing the expression of α-SMA, COL1, and FN induced by TGF-β1 through a mechanism regulated by p38 MAPK, which blocks the phosphorylation of GSK3β and decreases levels of active (unphosphorylated) β-catenin in the cytoplasm and nucleus [63,64,65]. Furthermore, inhibition of p38-MAPK also restores the sensitivity of myofibroblasts to apoptosis by inhibiting the ROS resistance induced by activating the antioxidant enzyme superoxide dismutase-1 [56]. In the liver, 15d-PGJ2 administration substantially attenuates hepatic inflammation and fibrosis by inhibiting phagocytic activity and reducing inflammatory cytokine expression in marrow-derived monocytes/macrophages [66]; this treatment also inhibits TGF-β-induced CTGF expression by preventing the phosphorylation of Smad2 through a mechanism independent of PPAR-γ [67]. Therefore, 15D-PGJ2 and its analogs may have clinical application value in a variety of fibrotic diseases due to the beneficial antifibrotic effect of 15D-PGJ2 and its stability, which is higher than that of PGD2.

BW245C is a DP receptor-specific agonist that mimics the function of PGD2. In a pulmonary fibrosis cell model, BW245C inhibited the TGF-β-induced proliferation of pulmonary fibroblasts but did not affect the synthesis of collagen or the differentiation of myofibroblasts. In a mouse model of pulmonary fibrosis established by bleomycin treatment, BW245C significantly ameliorates the accumulation of inflammatory cells and collagen in the lungs [60]. In cardiac fibrosis models, rosiglitazone, a PPAR-γ receptor ligand, inhibits Ang II-induced myocardial fibroblast proliferation and the expression of plasminogen activator inhibitor-1, type I collagen, type III collagen, and fibronectin in vitro via interactions between PPAR-γ and the TGF-β1/Smad2/3 and JNK signaling pathways. Rosiglitazone also inhibits Ang II-induced ECM deposition in the left atrium of rats [68].

However, in a study of renal fibrosis, the PGD2–CRTH2 pathway was identified as a profibrotic factor for tubulointerstitial fibrosis and advanced renal failure. The urinary excretion of L-PGDS increases during the progression of renal disease, including the early stage of diabetic nephropathy and hypertension without any renal injury, which may indicate renal injury in these patients. Hideyuki et al. found that the L-PGDS-PGD2-CRTH2 pathway mediates the activation of Th2 lymphocytes to promote fibrosis in the renal cortex after UUO via the production of IL-4 and IL-13 [69]. In conclusion, the antifibrotic effect of PGD2 is also related to the receptor type.

### 4.3. PGI2

PGI2, also called prostacyclin, is an important physiological regulator of platelet aggregation, leukocyte adhesion, the proliferation and relaxation of vascular smooth muscle cells, and vascular homeostasis, and is primarily synthesized in endothelial cells [8,13].

#### 4.3.1. Production of PGI2

PGI2 is generated by the conversion of PGH2 catalyzed by PGIS, which belongs to the family of cytochrome P450 enzymes [8]. Various studies have highlighted the importance of PGIS expression in preventing fibrotic progression. For example, hypermethylation of the PGIS promoter mainly induced by DNMT1 and DNMT3b contributes to the downregulation of PGIS in hepatic fibrosis, increasing HSC activation and the expression of collagen I and α-SMA. The overexpression of PGIS in vivo or in vitro in HSCs inhibits cell activation and promotes apoptosis [70]. This outcome indicates that PGIS plays a pivotal role in fibrotic progression, and epigenetic modification is also involved in these pathological processes. Both of these factors are considered new targets for antifibrosis research. PGI2 is unstable below pH 8.0 (the half-life is 3 min at pH 7.4 and 37 °C) and is rapidly hydrolyzed to the stable compound 6-keto-PGF1α [8]. Therefore, the developed stable analogs of PGI2 are potential candidates that reproduce its biological activities.

The effects of PGI2 are mediated by the activation of the prostaglandin I receptor (IP) coupled with the Gs-type G protein, which activates intracellular cAMP signaling. Studies using IP−/− mice showed that cardiac hypertrophy, cardiomyocyte hypertrophy, and cardiac fibrosis were significantly greater in these animals than in wild-type mice, indicating that the IP receptor suppresses the development of pressure overload induced cardiac hypertrophy [71]. Endogenous peroxisome proliferator-activated receptor (PPAR)-α is known to be a potential nuclear receptor for PGI2 [72], which is also related to the antifibrotic activity of PGI2. The induction of PGI2 further activates PPAR-α [73]. Several studies have indicated that activation of PPAR-α by agonists prevents myocardial fibrosis and renal fibrosis in mice [74,75].

#### 4.3.2. Effect of PGI2 on Fibrosis

Since PGI2 is mainly involved in the regulation of vascular function, studies of the effect of PGI2 on fibrosis mostly focus on heart and renal diseases. More clearly, PGI2 acts as an antifibrosis agent. Myocardial PGI2 release is increased in dogs with cardiac hypertrophy [76], which may be related to the increased expression of the COX-2 gene in this disease state. Myocardial fibroblasts are an important source of PGI2 production, and the PGI2 produced by these cells not only acts on fibroblasts to inhibit the synthesis of ECM [77] but also acts on IP receptors of cardiomyocytes to inhibit cardiomyocyte hypertrophy [71].

Due to the instability of PGI2 with a half-life of 3 min at pH 7.4 and 37 °C, PGI2 analogs or IP receptor agonists are often used in fibrosis research instead of exogenous PGI2. According to several reports, PGI2 analogs or IP receptor agonists exert antifibrotic effects on different organs, such as the heart, lung, kidney, and pancreas, through multiple mechanisms. With the development of research, multiple PGI2 analogs, including beraprost, cicaprost, and iloprost, have been discovered and used in antifibrosis research. In the heart, both receptors of IP and PPAR are abundant in cardiac fibroblasts. Beraprost, a prostacyclin analog, inhibits cardiac fibroblast proliferation by activating IP but not PPAR, which might be related to a suppressive TGF-β/Smad pathway [78]. Moreover, beraprost sodium exerts a suppressive effect on kidney fibrosis by improving damaged peritubular capillaries, inhibiting inflammation and oxidative stress and subsequently relieving EndoMT and ECM deposition in mice [79]. Cicaprost, another prostacyclin analog, also inhibits the PDGF induced proliferation of noncardiomyocytes via activation of the IP receptor [71]. All of these results suggest that the PGI2 analogs described above inhibit fibrosis of the heart or kidney by activating IP or PPAR-α. However, PGI2 analogs have also been shown to reverse the fibrosis process that has already developed. Inhaled iloprost improves right ventricular function and reverses established right ventricular fibrosis partially by preventing collagen synthesis and by increasing collagen degradation via two complementary mechanisms: inhibiting the expression of CTGF to decrease the activation of cardiac fibroblasts and inducing the activation of MMP9 to degrade the matrix protein [80]. This drug also inhibits pulmonary fibrosis induced by bleomycin and is more effective at decreasing fibrotic changes than methylprednisolone [81]. In addition, ONO-1301 is another PGI2 analog that is more likely to be used in the clinic than others. ONO-1301, which lacks typical prostanoid structures, thus leading to improved biological and chemical stability accompanied by long-lasting prostacyclin activity and thromboxane synthase inhibitory activity, has become a popular IP receptor agonist for research. In vitro, ONO-1301 suppresses the TGF-β-induced cardiac fibroblast-to-myofibroblast transition and fibroblast proliferation and migration via the activation of IP. ONO-1301SR, a sustained-release form of ONO-1301, exerts the same effects accompanied by the downregulation of fibrosis-related cytokines (α-SMA, ECM, and TGF-β) and upregulation of cardioprotective cytokines hepatic growth factor (HGF), vascular endothelial growth factor (VEGF), and stromal cell-derived factor 1 (SCDF-1) in a mouse transverse aortic constriction model [82]. Similar to the effect on the heart, ONO-1301MS may relieve the inflammation and remodeling that occur in individuals with asthma by suppressing airway hyperresponsiveness, allergic inflammation, and the development of remodeling in a chronic house dust mite-induced asthma model [83]. ONO-1301 attenuates pancreatic fibrosis by inhibiting monocyte activity not only through the induction of HGF but also through direct effects of ONO-1301 itself on a rat model of dibutyltin dichloride-induced chronic pancreatitis [84]. Moreover, ACT-333679, a selective IP receptor agonist, suppresses TGF-β1-induced myofibroblast transdifferentiation, proliferation, ECM synthesis, and IL-6 and plasminogen activator inhibitor-1 secretion via the activation of cAMP-induced YAP/TAZ nuclear exclusion and subsequent suppression of YAP/TAZ-dependent profibrotic gene transcription [85].

PGI2 negatively regulates the fibrotic response of cells in various tissues by activating the corresponding receptors. However, the instability of PGI2 and its analogs limits their application. The discovery or synthesis of additional receptor agonists with non-PGI2 structures will have strong clinical application prospects.

### 4.4. PGF2α

#### 4.4.1. Production of PGF2α

PGF2α is produced from PGH2 by PGFSs that are present in almost all tissues. PGF2α produced by these enzymes plays multiple and important roles in the female reproductive system, regulating oogenesis, ovulation, luteolysis, contraction of uterine smooth muscle, and initiation of parturition [8,13]. In addition to its actions in the reproductive system, PGF2α also mediates processes in the kidney, contraction of arteries, myocardial dysfunction, brain injury, and pain [13]. Due to their ability to decrease intraocular pressure, PGF2α derivatives, such as latanoprost, bimatoprost, and travoprost, are first-line drugs for the treatment of glaucoma worldwide [86].

Three types of PGFS have been identified, PGH 9,11-endoperoxide reductase, PGD 11-ketoreductase, and PGE 9-ketoreductase, which catalyze the formation of PGF2α from PGH2, PGD2, and PGE2, respectively. However, PGD 11-ketoreductase converts PGD2 to 9α,11β-PGF2α, a PGF2α stereoisomer [8,87]. These enzymes function in the presence of NADH or NADPH, and PGD 11-ketoreductase and PGE 9-ketoreductase are members of the aldo-keto reductase (AKR) superfamily [88].

PGF2α binds to the receptor FP, which couples with the Gq protein to induce the production of intracellular inositol phosphates (IPs) that in turn increase the intracellular Ca^2+^ level by promoting its release from the endoplasmic reticulum of cells and activating PKC. In addition, other protein kinases, including MAPK and Rho kinase, are activated [89,90]. Two different splice variants of the FP receptor named FPA and FPB have been identified, which differ from each other in the length of the C-terminal tails [13]. Binding between PGF2α and the FP receptor is not completely specific. Reports have shown that PGF2α binds to EP1 and EP3 receptors with significant affinity, and some effects of PGF2α may be mediated by an EP receptor [22]. Moreover, the FP receptor binds PGD2 and PGE2 with EC50 values in the nanomolar range [13]. The human FP receptor is widely expressed in the lung tissue and is an attractive target for the treatment of fibrotic lung diseases [90].

Endogenous PGF2α is swiftly degraded in various organs to 13,14-dihydro-15-keto PGF2α (15-keto-dihydro PGF2α), a stable metabolite of PGF2α with a longer half-life in the circulation that has been used as a reliable indicator of PGF2α biosynthesis in vivo [91].

#### 4.4.2. Effect of PGF2α on Fibrosis

After several years of research, PGF2α was shown to exert a relatively clear effect on the process of fibrosis, promoting the development of fibrosis without involving TGF-β. In the lung, PGF2α is abundant in the bronchoalveolar lavage fluid of subjects with IPF [92], and the concentrations of 15-keto-dihydro PGF2α (a stable degraded form of PGF2α) are increased in the plasma of patients with IPF; these values correlate with indices of disease severity and prognosis in patients with IPF [91]. PGF2α/FP signaling induces pulmonary fibrosis independently of TGF-β by promoting fibroblast proliferation and collagen production via FP activation [92]. Additionally, the binding activates the small GTPase Rho signaling pathway, leading to collagen synthesis of the lung fibroblast [93]. In addition, PGF2α/FP receptor activation facilitates the pathogenesis of myocardial fibrosis in individuals with diabetic cardiomyopathy, accompanied by elevated cholesterol, triglyceride, glucose, and insulin levels, increased collagen deposition, and severe insulin resistance by activating PKC/Rho pathways, while FP receptor gene silencing alleviates myocardial fibrosis mainly by inhibiting this process [94,95]. Systemic sclerosis affects the skin and internal organs, leading to fibrosis. α2-Antiplasmins (α2AP) activate calcium-independent PLA2 through adipose triglyceride lipase and then promote PGF2α synthesis, which induces the expression of TGF-β and the development of dermal fibrosis in mice [96].

Because PGF2α clearly promotes fibrosis, antagonizing the FP receptor may be an antifibrotic strategy for the treatment of fibrosis. BAY-6672 is a novel synthetic antagonist that was first reported to exert beneficial effects on preclinical animal models of silica-induced pulmonary fibrosis, and it is expected to be a new therapeutic approach for IPF [90]. OBE022 is the only FP receptor antagonist currently in clinical development for the treatment of preterm labor [89]. Other FP receptor antagonists, including PGF2α dimethylamine, phloretin, gliben, AL-3138, AL-8810, THG113, PDC31, PDC113.824, and AS604872, have been discovered; However, their functions in fibrotic diseases remain unclear, and further research into their effects on fibrotic disease is needed in the future.

### 4.5. TXA2

TXA2 is another important AA metabolite that is mainly produced and secreted by platelets and is involved in regulating multiple physiological and pathological functions, such as platelet accumulation, smooth muscle contraction, allergies, modulation of acquired immunity, atherogenesis, neovascularization, and metastasis of cancer cells, by binding to its receptor [97]. In addition to platelets, macrophages, monocytes, neutrophils, and the lung parenchyma are also sources of TXA2 [98]. However, due to its instability, with a half-life of 30 seconds, TXA2 is often nonenzymatically degraded into another stable but inactive form of TXB2 [13].

#### 4.5.1. Production of TXA2

TXA2 is produced from PGH2 through a reaction catalyzed by thromboxane synthase (TXAS), a ferrihemoprotein and a CYP enzyme [8]. TXAS deficiency improves the effects of insulin and attenuates adipose tissue fibrosis by decreasing the expression and deposition of fibrotic collagens (Col1 and Col3) [99].

The TP receptor is the cognate receptor of TXA2, which belongs to the transmembrane G protein-coupled receptor family. The G proteins coupled with TP receptors mainly include Gq, G13, and multiple small G proteins (Gs and Gh), among which the activation of Gq leads to increased cytosolic Ca^2+^ concentrations that activate PKC; activation of G13 activates Rho kinase and is involved in the physiological response mediated by the Rho signaling pathway [97]. TP receptors are identified as two isoforms, TPα and TPβ, which are different from each other in the C-terminal region and have different actions. According to a previous study, TPα and TPβ exert opposite effects on activating adenylate cyclase, leading to an increase and decrease in the level of cAMP due to the coupling of Gs and Gi, respectively [100]. The expression of TPα on the cell surface is greater than that of TPβ, which is related to the hydrolysis of the TPβ C-terminal domain by a protease [101].

#### 4.5.2. Effect of TXA2 on Fibrosis

TXA2 has long been considered a proinflammatory and profibrotic lipid mediator in humans. In both human and murine platelets, the production of TXA2 is associated with COX-1 activity. COX-1 deletion in mouse platelets along with a deficiency of TXA2 highlights the role of platelet-derived TXA2 in the development of colitis and fibrosis induced by epithelial damage by inhibiting the proliferation and migration of myofibroblasts [102]. Due to the profibrotic effect of TXA2, various TP receptor antagonists have been used in antifibrosis research. For example, KP-496 and NTP42, TP antagonists that suppress acute or chronic lung inflammation and pulmonary fibrosis by inhibiting mast cell recruitment and pulmonary collagen deposition, may be expected to be new therapeutic agents for lung diseases characterized by inflammation and fibrogenesis, such as IPF and chronic obstructive pulmonary disease [103,104]. Moreover, the selective TP antagonist terutroban significantly prevents both TGF-1β and HSP47 expression, both of which play an important role in the onset and progression of various fibrotic diseases, to inhibit collagen deposition in the aortic wall of salt-loaded spontaneously hypertensive stroke-prone rats (SHRSPs) [105].

Based on the results described above, either inhibiting the production of TXA2 or antagonizing the function of the TP receptor reverse the profibrotic effect of TXA2. However, the inhibition of TXA2 synthesis through either of the pathways described above is widely accepted to shift the enzymatic conversion of the common precursor endoperoxide substrates PGG2/PGH2 away from TXA2 biosynthesis towards the generation of PGI 2 [104]. Therefore, we hypothesize that the antifibrotic effect produced by inhibiting TXA2 may also be partially mediated by PGI2.

## 5. Other Therapeutic Modalities Exert Antifibrotic Effects by Altering Endogenous PG Expression

Due to the unstable chemical properties of various PGs, exogenous drug delivery methods are limited. Therefore, increasing/decreasing endogenous PG expression using other approaches are also important strategies for the treatment of fibrosis. As mentioned above, PGE2, PGD2, and 15D-PGJ2 all inhibit the progression of fibrotic disease, and thus treatments designed to alter their production and degradation will achieve the same antifibrotic effect.

Mesenchymal stem cells (MSCs) are a type of pluripotent cell that has the ability to differentiate into other functional cells, such as epithelial cells and endothelial cells, to promote tissue regeneration and are currently well used in clinical studies related to various degenerative and/or inflammatory diseases [106]. One study revealed that an infusion of human adipose-derived MSCs inhibits the development of radiation-induced lung fibrosis and preserves the architecture of the irradiated lung, as represented by a lack of transformation of fibroblasts into myofibroblasts and reduced ECM formation within injured sites. This function is achieved by increasing the levels of the endogenous anti-fibrosis substances hepatocyte growth factor (HGF) and anti-fibrosis lipid PGE2 in serum and bronchoalveolar lavage fluid [107]. Although MSCs are quickly metabolized after injection, the antifibrotic effects persist for a long time. In addition to the function of MSCs in promoting the release of antifibrosis factors, they have also been used as gene carriers to participate in gene therapy. For example, MSCs modified with HGF or the TGF-β type II receptor (TβR) gene migrate to the site of lung injury and then release a large amount of HGF or TβR. HGF inhibits the fibrosis process by reducing the expression of proinflammatory factors and profibrotic proteins [108], and the released TβR neutralizes TGF-β in lung tissues, thereby blocking the profibrotic signaling pathway induced by TGF-β [109]. Therefore, MSCs can also be used to transfer PG-related genes, such as related PG synthases or receptors, to enhance/neutralize the role of PGs in target tissue and provide new ideas for the treatment of related fibrotic diseases.

Exosomes are also a hot topic in current research and have been used as a new therapeutic approach in the treatment of various diseases. Exosomes are an important structure containing a variety of active substances that transmit signals between cells, such as mRNAs and microRNAs. Some stimulating factors, such as inflammatory factors and cytokines, induce target cells to release exosomes and transmit signals between cells. IL-1β induces and activates human lung fibroblasts to secrete a large number of PGE2-containing exosomes. PGE2 contained in exosomes is delivered to the receptors expressed on fibroblast or epithelial cell surfaces and then exert its antifibrotic effects by activating EP2/EP4, which is also the mechanism by which IL-1β exerts its antifibrotic effects [110]. Moreover, exosomal transmission of information can concentrate a large number of active PGE2 molecules on the surface of target cells, prolong the action time of PGE2, strengthen the activation of receptors, and thus exert a stronger antifibrotic effect.

As described above, 15-PGDH regulates the degradation of PGs. Due to the extensive antifibrotic effect of PGE2, many studies have focused on its degradation pathway, especially the enzyme 15-PGDH, to evaluate its role in fibrotic diseases. Pulmonary endothelial cells, macrophages, and mast cells express PGDH, which is the direct cellular target of PGDH inhibitors [111], and 15-PGDH is expressed at high levels in the residual alveolar tissues of patients with IPF compared with normal individuals [30].Inhibition of this enzyme decreases alveolar epithelial cell apoptosis, fibroblast proliferation and fibrocyte differentiation, reduces collagen production in IPF precision-cut lung slices and in the bleomycin model, inhibits inflammatory pathology, and subsequently improves the lung function of a mouse bleomycin model by increasing the production of PGE2 [30,111]. TD88, as a 15-PGDH inhibitor based on the thiazolinedione structure, prevents the excessive accumulation of collagen and improves the re-epithelization of a dermal wounded surface due to the downregulated expression of PDGF, CTGF, and TIMP-2 mediated by PGE2 [112]. These findings highlight the role of 15-PGDH in IPF and skin wound healing, suggesting that 15-PGDH inhibition is a promising therapeutic approach.

In conclusion, in addition to the application of exogenous PG analogs or receptor agonists/antagonists, the aforementioned approach to alter the level of resistant PGs in vivo undoubtedly has new prospects for inhibiting fibrosis development. Moreover, we have concluded in the previous chapter that PGE2 is able to reverse already formed IPF. The fibrosis process is easier to reverse by changing the endogenous PGE2 level using the methods described above, and these treatments are safer and are not affected by the chemical properties of PGE2.

## 6. Conclusions and Perspectives

In this review, we summarized the roles of PGs in different fibrotic models involving PG synthases, specific receptors, corresponding analogs or receptor agonists/antagonists, and the downstream signaling participating in these processes.

**Figure 2:**
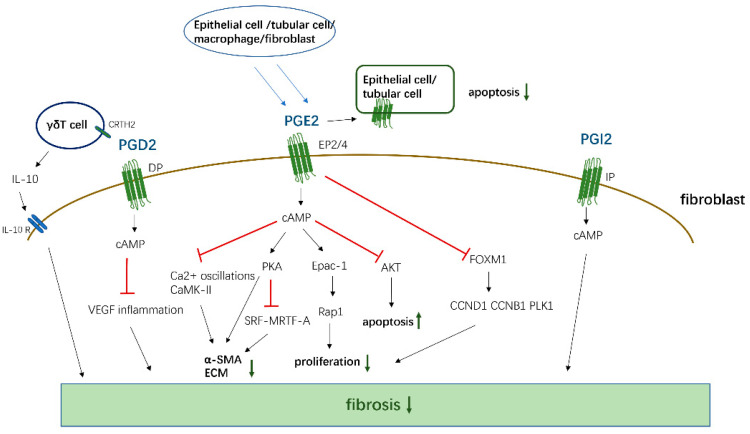
The antifibrotic effects of PGD2, PGE2, and PGI2. PGD2, PGE2, and PGI2 activate cAMP by binding to corresponding receptors, and then inhibit the proliferation, transformation, and ECM generation of fibroblasts, and promote cell apoptosis by affecting downstream signals. At the same time, PGD2 also acts on the CRTH2 receptor on γδT cell, promoting its release of anti-fibrosis IL-10 to inhibit fibrosis. PGE2 can act on EP2/4 receptors in epithelial cells and Tubular Cells in an autocrine way, thereby inhibiting the apoptosis of these functional cells.

**Figure 3:**
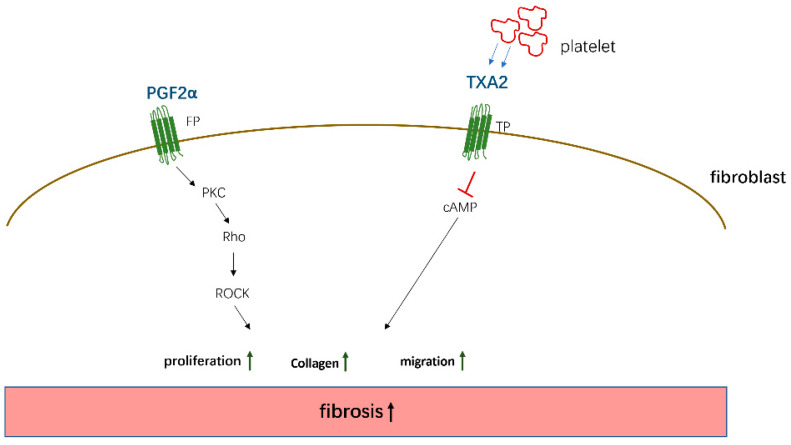
The pto-fibrotic effects of PGF2α and TXA2. PGF2α acts on the FP receptor on the surface of fibroblasts to induce cell proliferation and collagen expression by activating PKC/Rho kinase—ROCK (Rho-associated kinase) pathway. TXA2 secreted by platelets acts on TP receptors and promotes fibrosis by activating cAMP signaling.

PGE2, PGD2, 15d-PGJ2, and PGI2 mainly mediate antifibrotic reactions by binding to their homologous receptors and activating downstream G protein-related kinase reactions along with the induction of cAMP synthesis, leading to the inhibition of proliferation and migration, transformation of interstitial cells such as fibroblasts into myofibroblasts, and the promotion of apoptosis to reduce the deposition of ECM proteins in various organs, which are summarized in Figure 2. Moreover, these PGs inhibit the apoptosis of parenchymal cells, thereby reducing organ structural failure and dysfunction. However, PGE2 and PGD2 also play a role in promoting fibrosis in certain tissues and cells. These contradictory findings are related to the type of receptors, EP1/EP3, and the cells studied. However, PGF2α and TXA2 exert opposite effects compared to the aforementioned PGs and mainly promote the occurrence of fibrotic reactions summarized in Figure 3. Through sorting, we found that the level of PGs changes in different disease states. For example, in patients with IPF, the levels of COX-2/PGE2 in alveolar lavage fluid are decreased, and the level of PGF2α is increased. Although these changes in PGs are different, the combined effect of these changes contributes to the development of fibrosis in patients.

Due to the instability of PGs, an increasing number of studies are now focusing on inhibiting synthetases or synthesizing corresponding receptor agonists/antagonists to mimic or antagonize the responses mediated by related PGs (Table 2). These agents will undoubtedly be able to simulate or block the response of PGs to the fibrosis process and provide additional alternative treatment methods for fibrotic diseases. Although many studies have described the effects of these PG analogs or receptor agonists/antagonists, most of these studies have focused on animal experiments, and few clinical studies have been conducted. In the future, relevant clinical trials may be needed to further determine the value of these PG analogs or receptor agonists/antagonists in the prevention or treatment of fibrotic diseases in humans. Moreover, in addition to the abovementioned drug therapy, we also summarized the positive effects of MSCs, exosomes, and 15-PGDH inhibitors on fibrotic diseases. By altering the levels of endogenous PGs, these novel therapies may better mimic the effects of PGs in vivo and have a higher safety profile. The emergence of these novel therapeutic modalities not only provides new targets for the treatment of the disease but also provides a new method to exogenously administer PGs and the abovementioned analogs and agonists/antagonists.

Moreover, regardless of whether PGs inhibited or promoted the response to fibrosis, the effect was mediated by the activation of the corresponding receptor and its downstream G protein, and different G proteins induced different effects. For example, the Gs protein is related to antifibrotic effects, while the Gq and Gi proteins promote fibrosis. In conclusion, if we can synthesize non-PG structural agonists, antagonists targeting PG receptors, or small-molecule compounds that stimulate G protein alone, these drugs will further promote the development of antifibrotic treatments with fewer side effects. Therefore, these molecules will be valuable in future clinical applications.

## Figures and Tables

**Table 1:** PG receptor associated signal transduction and effect on fibrosis.

Prostaglandins	Receptors	G Proteins	Second Messengers	Fibrosis
PGE2	EP1	Gq	IP3, ↑ Ca^2+^ ↓	+
	EP2	Gs	cAMP ↑	−
	EP3	Gi, G12, GRho	camp, ↓ Ca^2+^ ↑	+
	EP4	Gs	cAMP ↑	−
PGD2	DP1	Gs	cAMP ↑	−
	DP2	Gi	cAMP ↓, Ca^2+^ ↑	+
PGI2	IP	Gs	cAMP ↑	−
	PPAR-α			−
PGF2α	FP: FPA FPB	Gq, GRho	IP3, ↑ Ca^2+^ ↑	+
TXA2	TP:TPα TPβ	Gq, G13 Gs, Gi, Gh	Ca^2+^ ↑ (Gq) cAMP ↑↓ (Gs Gi)	+

“↑” stands for ascending, “↓” for descending, “+” for promoting fibrosis, and “−” for inhibiting fibrosis.

**Table 2:** Effects of analogs or receptor agonists/antagonists of PGs on fibrosis.

Name	Experimental Subjects	Functions and Mechanisms
Butaprost (EP2 receptor agonist)	Madin-Darby canine kidney cells/ unilateral ureteral obstruction mouse model/ human precision-cut kidney slices	Reduce the expression of α-SMA, fibronectin, and col1 by inhibition of TGF-β/Smad signaling pathway [38,40]
ONO-0260164 (EP4 receptor agonist)	cardiac fibroblasts/ cardiac hypertrophy mouse model	Downregulate collagen type 1 and type 3 through PKA activation [51]
BW245C (DP receptor agonist)	pulmonary fibroblasts/ bleomycin induced pulmonary fibrosis mouse model	Inhibit TGF-β-induced proliferation of fibroblasts in vitro; ameliorate the accumulation of inflammatory cells and collagen in the lungs in vivo [60]
Rosiglitazone (PPAR-γ receptor agonist)	myocardial fibroblast/ Ang II-infused rats	Suppress Ang II-induced production of PAI-1 and ECM via interactions between PPAR-γand TGF-β1/Smad2/3 and JNK signaling pathways [68]
Beraprost, (PGI2 analog)	cardiac fibroblast	Inhibit cardiac fibroblast proliferation by activating IP via suppressing TGF-β/Smad pathway [78]
	HUVECs/ Kidney UUO mouse model	Improve damaged peritubular capillaries, inhibit inflammation and oxidative stress, reliev EndoMT and ECM deposition in mice [79]
Cicaprost, (PGI2 analog)	noncardiomyocyte	Inhibit proliferation of noncardiomyo-cytes via activation of the IP receptor in vitro [71]
Iloprost (PGI2 analog)	cardiac fibroblasts/ Angio-obliterative pulmonary arterial hypertension and right ventricular(RV) failure rat model	Improve RV function and reverse established RV fibrosis by preventing collagen synthesis and increasing collagen degradation [80]
	bleomycin induced pulmonary fibrosis rat model	inhibit pulmonary fibrosis [81]
ONO-1301 (PGI2 analog) ONO-1301SR/ONO-1301MS (sustained-release form of ONO-1301)	cardiac fibroblast/ transverse aortic con-striction mouse model	Suppress TGF-β-induced fibroblast proliferation, migration and myofibro-blast transition Downregulate fibrosis-related cytokines α-SMA, ECM, and TGF-β, upregulate cardioprotective cytokines HFG, VEGF, SCDF-1 [82]
	chronic dust mite-induced asthma house model	Suppress airway hyperresponsiveness, allergic inflammation, the development of remodeling [83]
	dibutyltin dichloride induced chronic pancreastitis rat model	Attenuate pancreatic fibrosis by inhibiting monocyte activity [84]
ACT-333679 (IP receptor agonist)	lung fibroblasts	Induce YAP/TAZ nuclear exclusion and suppress YAP/TAZ-dependent profibrotic gene transcription via activation of cAMP [85]
BAY-6672 (FP receptor antago-nist)	silica-induced pulmonary fibrosis model	Exert beneficial effects via antagonizing FP [90]
KP-496 (dual antagonist of the cysLTs and TXA2 receptors)	bleomycin induced pulmonary fibrosis mouse model	Decrease the numbers of macrophages, neutrophils, eosinophils and hydroxyl-L-proline content in BALF [103]
NTP42 (TP receptor antago-nist)	monocrotaline induced pulmonary arterial hypertension rat model	Reduce pulmonary vascular remodelling, inflammatory mast cell infiltration and fibrosis [104]
Terutroban (selective TP receptor antagonist)	SHRSPs	Prevent expression of TGF-1β and HSP47 [105]
